# Mortality patterns and risk among older men and women with intellectual disability: a Swedish national retrospective cohort study

**DOI:** 10.1186/s12877-017-0665-3

**Published:** 2017-11-22

**Authors:** Nawi Ng, Eva Flygare Wallén, Gerd Ahlström

**Affiliations:** 1 Unit of Epidemiology and Global Health, Department of Public Health and Clinical Medicine, Faculty of Medicine, Umeå University, SE-901 87 Umeå, Sweden; 2 Centre for Demographic and Ageing Research, Umeå University, SE-901 87 Umeå, Sweden; 3 Karolinska Institutet (KI), Department of Neurobiology, Care Sciences and Society (NVS), Division of Family Medicine and Primary Care, Alfred Nobels allé 23, D2, SE-141 83 Huddinge, Sweden; 4 Department of Health Sciences, Faculty of Medicine, Lund University, P.O. Box 157, SE-221 00 Lund, Sweden

**Keywords:** Intellectual disability, Mortality risk, Cause of death, Retrospective cohort study, Sweden

## Abstract

**Background:**

Sweden has closed all institutions and imposed legislation to ensure service and support for individuals with intellectual disability (ID). Understanding mortality among older individuals with ID is essential to inform development of health promotion and disease control strategies. We investigated patterns and risk of mortality among older adults with ID in Sweden.

**Methods:**

This retrospective cohort study compared older adults aged 55 years and older with ID with a control population. Participants were followed during 2002–2015 or death, and censored if they moved out of Sweden. Individuals with ID were identified from two national registers: one covering all specialist health-care visits (out-patient visits and hospitalisation) and the other covering people accessing social/support services. Individuals with ID (*n* = 15,289) were matched with a control population by sex, birth year, and year of first hospitalisation/out-patient visit/access to LSS services. Cause-of-death data were recorded using International Classification of Diseases, Tenth Revision. Cox proportional hazards regression were conducted to assess if overall and cause-specific mortality rate among individuals with ID was higher than in the Swedish population.

**Results:**

The overall mortality rate among individuals with ID was 2483 per 100,000 people compared with 810 in the control population. Among those who died, more individuals with ID were younger than 75 years and unmarried. Leading causes of death among individuals with ID were circulatory diseases (34%), respiratory diseases (17%) and neoplasms (15%). Leading causes of death in a sub-sample with Down syndrome (DS) were respiratory diseases (37%), circulatory diseases (26%) and mental/behavioural disorders (11%). Epilepsy and pneumonitis were more common among individuals with ID than controls. Alzheimer’s disease was common in the control population and individuals with DS, but not among those with ID when DS was excluded. Individuals with ID had a higher overall mortality risk (hazard ratio [HR] 4.1, 95% confidence interval [CI] 4.0–4.3) and respiratory disease death risk (HR 12.5, 95% CI 10.9–14.2) than controls.

**Conclusion:**

Older adults with ID in Sweden carry a higher mortality risk compared with the general population, mainly attributable to respiratory, nervous and circulatory diseases. Care for this group, particularly during the terminal stage of illness, needs to be tailored based on understanding of their main health problem.

**Electronic supplementary material:**

The online version of this article (10.1186/s12877-017-0665-3) contains supplementary material, which is available to authorized users.

## Background

As in the general population, life expectancy among people with intellectual disability (ID) has increased in recent decades [1, 2]. The median age of Swedes with Down syndrome (DS) increased by 1.8 years annually from 1969 to 2003, with a median age at death of almost 60 years in 2003 [3]. Excluding those with severe and multiple disabilities or DS, the gap in life expectancy between people with less severe ID and the general population is decreasing [1, 2, 4]. Globally, life span, health and disease patterns and causes of death among individuals with ID remain under-studied at a national level [5]. Research on mortality among individuals with ID in Sweden is scarce. No systematic review and/or meta-analysis on the cause of death among older population with intellectual disabilities exist. The few relevant studies identified in PubMed were conducted among children/adults and among small samples at a sub-national/county level [3, 6–10].

In Sweden and other Nordic countries, living conditions for individuals with ID have undergone significant changes in the past 50 years. Older individuals with ID have experienced moving from institutions to group homes and community care [11]. The ‘normalisation principle’ aims to provide individuals with ID daily living patterns and conditions that are as close as possible to ‘norms and patterns of the mainstream of society’ [12]. In Sweden, normalisation went through several stages, reflected in changes in policy and legislation. The right-based LSS legislation, an entitlement law that guarantees good living conditions for people with extensive and permanent functional impairment, came into force in 1994 [13]. The LSS Act complements the Social Services Act (SoL), which regulates how social services function in the general population [14].

Studies on morbidity and cause of death in the population with ID vary in terms of the population studied, study design, and data sources analysed. Hence, it is difficult to draw conclusions regarding health patterns and leading causes of death among people with different ID diagnoses [1]. There is evidence that individuals with ID age at an earlier chronological age and have more degenerative diseases compared with the general population [15–17]. Individuals with ID also have a higher prevalence of mental health problems [18, 19]. A longitudinal study in Finland reported cardiovascular and respiratory diseases (mainly pneumonia) as the two leading causes of death in a population with ID [20], and lower mortality rate from neoplasms and external causes compared with the general population [20]. Therefore, older adults with ID may need different preventive healthcare programmes and have different healthcare use. There are also concerns about the lack of medical expertise in the healthcare and community care systems, which might result in sub-standard and less optimal care for individuals with ID [4, 8, 21, 22].

A report published by the Swedish National Board of Health and Welfare in 2010 described unequal living conditions for adults with functional impairments in Sweden [23]. Except for the housing standard, people with functional impairments (defined as people with support from LSS services and care under the SoL) were mainly older, had psychiatric diseases, and had worse living conditions than the general population. Individuals with ID represent a more vulnerable group because of their cognitive and physical impairments and dependency on others as caregivers. The report described individuals with ID as having a low education level, being frequently excluded from the labour market and unemployed, having weak personal finances and less active leisure time. The use of psycho-pharmacological drugs was three times higher among individuals with ID, autism, or autism-like conditions than in the general population [23]. Similar health inequalities among individuals with ID have been reported in other European countries [15].

To date, there is no single national register covering all individuals diagnosed with ID. The national LSS register covers all individuals with ID who receive service and support under LSS legislation [13]. The National Patient Register (NPR) contains information about individuals with ID if the ID diagnoses were registered in contacts with the healthcare system. An ID diagnosis is not automatically mentioned on a death certificate [24]. Most studies involving the population with ID have compiled information from different registers to estimate the national prevalence of ID, health patterns and leading causes of death [25–27].

Older individuals with ID in Sweden were born before the closure of care institutions in the 70s, and have lived with full implementation of the LSS law during at least one decade. The living circumstances of individuals with ID and their life expectancy have also changed considerably in recent decades. In light of these positive societal developments and changes in living conditions, it is desirable to gain knowledge about ‘first generation older people with ID’ who experienced this transition, their ageing process and health risks, and whether their health differs from that of older people in general. The objectives of this study were: (i) to investigate the patterns of mortality and causes-of-death among older men and women with ID in Sweden compared with a matched control population from 2002 to 2015; and (ii) to estimate the risk of death of older men and women with ID compared with the matched control population.

## Methods

### Design

This study used a retrospective cohort design, in which we constructed two cohorts: older men and women aged 55 years and older with ID from two national registers, and a matched control population drawn from the population register. The cohorts were followed up during 2002–2015 to observe subsequent mortality as recorded in the death register.

### Identification of individuals in datasets

Information about individuals with ID in Sweden is available from two national registers: (i) the NPR and (ii) the LSS register based on the Swedish Act concerning Support and Service for Persons with Certain Functional Impairments [13]. The Swedish National Board of Health and Welfare maintains both registers.

The NPR includes all inpatient hospital admissions since 1987 and outpatient care since 1992. It is based on mandatory registration for all 21 county councils in Sweden. In this study, we identified all older adults aged 55 years or older who were recorded in the NPR from 2002 to 2015 as having a diagnosis of ID. ID diagnoses were based on the International Classification of Diseases, Tenth Revision (ICD-10), and included the diagnosis categories: mental retardation (F70–73, F78–79), disorders of psychological development (F84, F88–89) and DS (Q90). We excluded diagnosis codes for F84.1 (atypical autism) and F84.5 (Asperger syndrome) to ensure a well-defined study group. Only the first event of hospitalisation was extracted for each individual. We recorded information on the year of hospitalisation, birth year, and sex.

The LSS register started in 2004 and is based on mandatory yearly registration from all 290 municipalities in Sweden. In 2007, the register started recording three different types of disability. In this study, we included all individuals with ID, autism and conditions resembling autism who had received any LSS services. We focused on all adults aged 55 or older who received LSS services from 2007 to 2015. As with the NPR, we only identified the first time an LSS service was used, and extracted information on the year of first access to LSS services, birth year, and sex.

**Fig. 1:**
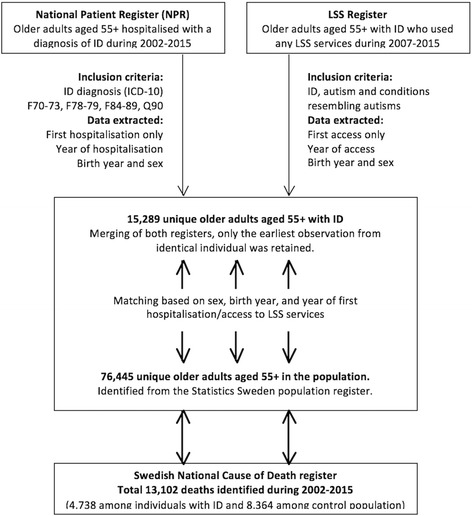
Identification of datasets and study subjects

Figure 1 shows how the two extracted datasets were merged into single dataset, resulting in 15,289 observations for unique individuals. Statistics Sweden matched each individual with ID in the dataset with five controls with the same sex, birth year, and year of first hospitalisation/access to LSS services, identified from the Swedish population register. All individuals were alive on January 1, 2002, the date chosen as the starting point of the observation. We calculated the age for all individuals in the dataset in 2002. The youngest individuals were aged 42 years in 2002 (they were either hospitalised or accessed LSS services when they were 55 years old in the last year of follow-up; i.e. 2015).

The dataset was linked with data from the Swedish National Cause of Death register, maintained by the National Board of Health and Welfare, to identify deaths among individuals included in the study from 2002 to 2015. Data extracted from this register and merged with the datasets described above were: death date, age at death, underlying/main cause of death, contributing causes of death, marital status, and country of birth (Swedish- or foreign-born). Marital status was coded as married or not married (including those who were single, divorced or widowed).

In total, 13,102 deaths (4738 among individuals with ID and 8364 in the control population) were identified for the 91,734 individuals included in the study (cohort with ID and control population). We recoded detailed cause-of-death data to obtain causes of death at an ICD-10-chapter level. For 604 deaths, the underlying/main cause of death was recorded as one of the ICD-10 diagnosis codes for ID: F70–73/F78–79, mental retardation (*n* = 180); F84–89, disorders of psychological development (*n* = 4); and Q90, DS (*n* = 420). As we did not regard ID as a cause-of-death, we replaced the underlying/main cause of death for these 604 individuals with diagnosis codes for contributing causes of death that were not the ID diagnosis codes specified above. No other contributing causes of death were available for 60 of the 604 individuals, and the cause-of-death data were recoded as an ‘unclassified diagnosis’ for these individuals.

The final dataset included 374 deaths (2.9% of all deaths) with an unclassified cause of death (166 deaths among individuals with ID and 208 deaths in the control population). DS was recorded as the cause of 942 deaths among individuals with ID (604 underlying as described above and 338 contributing cause of death). The remaining 7422 deaths among individuals with ID were attributed to other causes.

### Analysis

We defined the follow-up period as from 1 January 2002 to either 31 December 2015 or the date of death for those who died during the study period (2002–2015). In total, 389 individuals moved out of Sweden during the study period and were censored. For 18 individuals without a specific date or month of death, we imputed the date of death as 1 July of the respective year. For 65 individuals without a specific date of death, we imputed the date of death as 15th of the respective month of death.

Differences in demographic characteristics and death-related data between the two groups were compared in a bivariate analysis using chi-square tests for proportions. Differences in the mean age of death and follow-up period (years) between the two groups were assessed using two-sample *t*-tests. We conducted a sub-sample analysis to assess differences in marital status and nationality between individuals who died in the two groups. We calculated the proportionate mortality (%) and cause-specific mortality rate (per 100,000 population) in each group and among individuals with DS. We estimated the cause-specific mortality rate ratio between individuals with ID and the control population using conditional fixed-effect Poisson regression. We conducted conditional Cox proportional hazards regression with a robust variance estimator to assess if overall and cause-specific mortality differed between the two groups. A *p*-value below 0.05 was considered statistically significant. All analyses were conducted using Stata Version 13 [28].

## Results

### Overall mortality and sociodemographic characteristics

The crude mortality rate was 2483 per 100,000 people among individuals with ID and 810 in the control population (Table 1). This yielded an unadjusted incidence rate ratio of 3.07 (95% confidence interval [CI] 2.96–3.18), indicating the mortality rate among individuals with ID was three times higher than in the control population. Individuals with DS had an 11-fold higher mortality risk than the control population (incidence rate ratio 10.6, 95% CI 9.86–11.5). They also died earlier compared with individuals with other ID diagnoses and the control population, with the mean age at death being 63.5 years in those with DS, 72.1 years in those with other ID diagnoses and 76.2 years in the control population. Only 5.7% of individuals with DS were aged 65 years and older compared with about 16% of the control population.

**Table 1:** Demographic characteristics of the two groups at baseline (2002) and mortality during the follow-up period (up to 2015)

	Individuals with intellectual disability (*n* = 15,289) n (%)			Matched control population (*n* = 76,445) n (%)
	Sub-sample of individuals with DS (*n* = 942) n (%)	Sub-sample of individuals with ID (DS excluded) (*n* = 14,347) n (%)	All individuals with ID (*n* = 15,289) n (%)	
Sex				
Men	486 (51.6)	7844 (54.7)	8330 (54.5)	41,650 (54.5)
Women	456 (48.4)	6503 (45.3)	6959 (45.5)	34,795 (45.5)
Age group in 2002				
42–44 year	88 (9.3)	1720 (12)	1808 (11.8)	9040 (11.8)
45–54 year	440 (46.7)	6044 (42.1)	6484 (42.4)	32,420 (42.4)
55–64 year	360 (38.2)	4182 (29.2)	4542 (29.7)	22,710 (29.7)
65–74 year	50 (5.3)	1755 (12.2)	1805 (11.8)	9025 (11.8)
75–79 year	1 (0.1)	401 (2.8)	402 (2.6)	2010 (2.6)
80+ year	3 (0.3)	245 (1.7)	248 (1.6)	1240 (1.6)
Mean age at death (standard deviation)*	63.5 (5.4)	72.1 (9.4)	70.7 (9.5)	76.2 (10.3)
Average years of follow-up (standard deviation)*	9.4 (4.0)	12.7 (2.7)	12.5 (2.9)	13.5 (1.7)
Number of deaths during 2002–2015 (%)	761 (80.8)	3977 (27.7)	4738 (31.0)	8364 (10.9)
Total follow-up period in person-year*	8848	181,940	190,788	1,032,245
Mortality rate during 2002–2015 (per 100,000 people)*	8600	2186	2483	810

*Mean age at death, mortality rate and total follow-up period differed significantly between the two groups (*p* < 0.001). The groups were matched by sex, birth year and year of first hospitalisation/access to LSS services

Table 2 shows the demographic characteristics of individuals who died in each group. Of all deaths that occurred among individuals with ID (*n* = 4738), about 87% were younger than 75 years, 96% were not married and only 2.8% were foreign-born. Almost all the sub-sample with DS were younger than 75 years and not married. Of deaths that occurred in the control population, three-quarters were younger than 75 years and 52% were married; 58% were men and 11% were foreign-born.

**Table 2:** Characteristics of deaths in the group with intellectual disability (ID) and the matched control population

	Deaths among individuals with intellectual disability (ID) (*n* = 3147)			Deaths among matched control population
	Sub-sample of individuals with DS (*n* = 761) n (%)	Sub-sample of individuals with ID (DS excluded) (*n* = 3977) n (%)	All individuals with ID (*n* = 4738) n (%)	Total (*n* = 8364) n (%)
Sex				
Men	385 (50.6)	2227 (56.0)	2612 (55.1)	4877 (58.3)
Women	376 (49.4)	1750 (44.0)	2126 (44.9)	3487 (41.7)
Age group in 2002				
42–44 year	18 (2.4)	36 (0.9)	54 (1.1)	51 (0.6)
45–54 year	344 (45.2)	852 (21.4)	1196 (25.2)	1172 (14.0)
55–64 year	346 (45.5)	1446 (36.4)	1792 (37.8)	2450 (29.3)
65–74 year	49 (6.4)	1067 (26.8)	1116 (23.6)	2501 (29.9)
75–79 year	1 (0.1)	339 (8.5)	340 (7.2)	1160 (13.9)
80+ year	3 (0.4)	237 (6.0)	240 (5.1)	1030 (12.3)
Marital status				
Married	9 (1.2)	195 (5.0)	204 (4.4)	4284 (51.6)
Not married	732 (98.8)	3743 (95.1)	4475 (95.6)	4025 (48.4)
Country of birth				
Swedish-born	749 (98.4)	3857 (97.0)	4606 (97.2)	7479 (89.4)
Foreign-born	12 (1.6)	120 (3.0)	132 (2.8)	885 (10.6)

The control population was matched by sex, birth year and year of first hospitalisation/access to LSS services to individuals with ID. All variables differed significantly between the two groups; that is, all individuals with ID and their matched control population (*p* < 0.001)

### Cause-specific mortality proportions and rates

Table 3 presents the cause-specific proportionate mortality (%) and cause-specific mortality rates for individuals with ID and the control population, and the mortality rate ratio (95% CI) for each cause. The leading cause of death in both groups was diseases related to the circulatory system (about 35% of all deaths in both groups). However, the mortality rate for diseases of the circulatory system was about three times higher among individuals with ID compared with the control population, with a mortality rate ratio of 2.71 (95% CI 2.55–2.87). Diseases of the respiratory system were the second most common cause of death among individuals with ID (17% of all deaths), with a mortality rate ratio of 7.61 (95% CI 6.82–8.49) compared with the control population. Although neoplasms accounted for 33% of all deaths and was the second leading cause of death in the control population, we observed a lower mortality rate for neoplasms in the control population compared with individuals with ID. The mortality rate ratios for all cause-specific mortality rates were significantly higher for individuals with ID than the control population, except for diseases related to the skin and subcutaneous tissue (*p* > 0.05). When analysed specifically for individuals with DS (*n* = 513) (Table 4), the three leading causes of death were related to diseases of the respiratory system, diseases of the circulatory system and mental and behavioural disorders.

**Table 3:** Causes of death among all individuals with intellectual disability and the matched control population

Causes of death	Deaths among individuals with intellectual disability (ID) (*n* = 4738)		Deaths among matched control population (*n* = 8364)		Adjusted cause-specific mortality rate ratio (95% CI)*
	Number of deaths n (%)	Cause-Specific Mortality Rate (per 100,000 people)	Number of deaths n (%)	Cause-Specific Mortality Rate (per 100,000 people)	
Ch. I – Certain infectious and parasitic diseases, A00-B99	181 (3.8)	95	170 (2.0)	16	5.32 (4.32–6.56)
Ch. II – Neoplasms, C00-D48	710 (15.0)	372	2749 (32.9)	266	1.29 (1.19–1.40)
Ch. III – Diseases of the blood and blood-forming organs and certain disorders involving the immune mechanism, D50-D89	25 (0.5)	13	21 (0.3)	2	5.95 (3.33–10.6)
Ch. IV – Endocrine, nutritional and metabolic diseases, E00-E90	174 (3.7)	91	228 (2.7)	22	3.82 (3.13–4.65)
Ch. V – Mental and behavioural disorders, F00-F99	224 (4.7)	117	342 (4.1)	33	3.27 (2.77–3.88)
Ch. VI – Diseases of the nervous system, G00-G99	296 (6.3)	155	325 (3.9)	31	4.55 (3.89–5.33)
Ch. IX – Diseases of the circulatory system, I00-I99	1601 (33.8)	839	2959 (35.4)	287	2.71 (2.55–2.87)
Ch. X – Diseases of the respiratory system, J00-J99	807 (17.0)	423	530 (6.3)	51	7.61 (6.82–8.49)
Ch. XI – Diseases of the digestive system, K00-K93	205 (4.3)	107	318 (3.8)	31	3.22 (2.70–3.84)
Ch. XII – Diseases of the skin and subcutaneous tissue, L00-L99	5 (0.1)	3	14 (0.2)	1	1.79 (0.64–4.96)
Ch. XIII – Diseases of the musculoskeletal system and connective tissue, M00-M99	30 (0.6)	16	40 (0.5)	4	3.75 (2.34–6.02)
Ch. XIV – Diseases of the genitourinary system, N00-N99	105 (2.2)	55	99 (1.2)	10	5.30 (4.03–6.98)
Ch. XVI - Certain conditions originating in the perinatal period, P00-P96	9 (0.2)	5	0 (0)	0	NA
Ch. XVII – Congenital malformations, deformations and chromosomal abnormalities, Q00-Q99	49 (1.0)	26	5 (0.1)	0.5	49.0 (19.5–123)
Ch. XVIII – Symptoms, signs and abnormal clinical and laboratory findings, not elsewhere classified, R00-R99	166 (3.5)	87	208 (2.5)	20	3.99 (3.25–4.89)
Ch. XIX – Injury, poisoning and certain other consequences of external causes, S00-T98	6 (0.1)	3	0 (0)	0	NA
Ch. XX – External causes of morbidity and mortality, V01-Y98	144 (3.0)	75	356 (4.3)	34	2.02 (1.67–2.45)

The control population was matched by sex, birth year and year of first hospitalisation/access to LSS services to individuals with ID. The total follow-up period for individuals with ID was 190,788 person-years, and for the control population was 1,032,245 person-years. The adjusted mortality rate ratio was obtained using conditional fixed-effects Poisson regression

**Table 4:** Underlying causes of death among individuals with Down syndrome (n = 513)

Causes of death	Number of deaths n (%)	Cause-Specific Mortality Rate (per 1000 people)
Ch. X – Diseases of the respiratory system, J00-J99	282 (37.1)	3187
Ch. IX – Diseases of the circulatory system, I00-I99	197 (25.9)	2227
Ch. V – Mental and behavioural disorders, F00-F99	81 (10.7)	916
Ch. VI – Diseases of the nervous system, G00-G99	59 (7.8)	669
Ch. I – Certain infectious and parasitic diseases, A00-B99	33 (4.3)	373
Ch. XVIII – Symptoms, signs and abnormal clinical and laboratory findings, not elsewhere classified, R00-R99	28 (3.7)	317
Ch. XI – Diseases of the digestive system, K00-K93	16 (2.1)	181
Ch. II – Neoplasms, C00-D48	15 (2)	170
Ch. IV – Endocrine, nutritional and metabolic diseases, E00-E90	14 (1.8)	158
Ch. XIV – Diseases of the genitourinary system, N00-N99	10 (1.3)	113
Ch. XVII – Congenital malformations, deformations and chromosomal abnormalities, Q00-Q99	9 (1.2)	102
Ch. XX – External causes of morbidity and mortality, V01-Y98	8 (1.1)	90
Ch. III – Diseases of the blood and blood-forming organs and certain disorders involving the immune mechanism, D50-D89	3 (0.4)	34
Ch. XIX – Injury, poisoning and certain other consequences of external causes, S00-T98	3 (0.4)	34
Ch. XIII – Diseases of the musculoskeletal system and connective tissue, M00-M99	2 (0.3)	23
Ch. XII – Diseases of the skin and subcutaneous tissue, L00-L99	0 (0)	NA
Ch. XVI - Certain conditions originating in the perinatal period, P00-P96	0 (0)	NA

The total follow-up period for individuals with Down syndrome was 8848 person-years

We further investigated the three leading causes of death within each selected major cause of death based on 3-digit ICD-10 chapters (Table 5). The analysis for neoplasms was stratified by sex to account for sex-specific reproductive tumours. Several causes of death, including heart failure, pneumonitis due to solids and liquids, Alzheimer’s disease and epilepsy, were more common among individuals with DS compared with the control population. Epilepsy was the underlying cause of death for 21.6% of all deaths related to the nervous system among individuals with ID (DS excluded), and accounted for 37% of deaths related to the nervous system among individuals with DS. The most common cause of death related to the nervous system in the control population was Alzheimer’s disease (44%), compared with 10% of individuals with ID (DS excluded) and 54% of individuals with DS. Overall, individuals with DS had higher cause-specific mortality rates compared with individuals with ID or the control population, particularly for deaths-related to heart failure, pneumonitis due to solids and liquids, Alzheimer’s disease and epilepsy (Additional file 1). The cause-specific mortality rate for Alzheimer’s disease was 215 per 100,000 population for individuals with DS, compared with 12 per 100,000 for individuals with ID and 14 per 100,000 for the control population.

**Table 5:** Three leading causes of death within each selected major cause of death, based on ICD-10 chapters

ICD categories	Underlying causes of death among individuals with DS (*n* = 761)	Underlying causes of death among individuals with ID (DS excluded) (*n* = 3977)	Underlying causes of death among matched samples from the control population (*n* = 8364)
Ch. IX – Diseases of the circulatory system (I00-I99)	*n* = 122	*n* = 1368	*n* = 2959
	I50 Heart failure (25.4%)	I21 Acute myocardial infarction (23.3%)	I21 Acute myocardial infarction (24.1%)
	I21 Acute myocardial infarction (14.8%)	I25 Chronic ischaemic heart disease (16.6%)	I25 Chronic ischaemic heart disease (21.3%)
	I25 Chronic ischaemic heart disease (12.3%)	I50 Heart failure (11.8%)	I50 Heart failure (7.7%)
Ch. X – Diseases of the respiratory system (J00-J99)	*n* = 51	*n* = 468	*n* = 530
	J69 Pneumonitis due to solids and liquids (31.4%)	J18 Pneumonia, organism unspecified (50.0%)	J44 Other chronic obstructive pulmonary disease (48.9%)
	J18 Pneumonia, organism unspecified (19.6%)	J44 Other chronic obstructive pulmonary disease (19.9%)	J18 Pneumonia, organism unspecified (24.5%)
	J45 Asthma (7.8%)	J69 Pneumonitis due to solids and liquids (10.0%)	J84 Other interstitial pulmonary diseases (8.7%)
	J20 Acute bronchitis (7.8%)		
	J98 Other respiratory disorders (7.8%)		
Ch. II – Neoplasms in men (C00-D48)	*n* = 9	*n* = 381	*n* = 1555
	D37 Neoplasm of uncertain or unknown behaviour of oral cavity and digestive organs (33.3%)	C18 Malignant neoplasm of colon (12.9%)	C34 Malignant neoplasm of bronchus and lung (18.3%)
	C25 Malignant neoplasm of pancreas (11.1%)	C61 Malignant neoplasm of prostate (10.5%)	C61 Malignant neoplasm of prostate (16.5%)
	C67 Malignant neoplasm of bladder (11.1%)	C34 Malignant neoplasm of bronchus and lung (7.1%)	C25 Malignant neoplasm of pancreas (6.8%)
	C16 Malignant neoplasm of stomach (11.1%)		
	C90 Multiple myeloma and malignant plasma cell neoplasms (11.1%)		
	C76 Malignant neoplasm of other and ill-defined sites (11.1%)		
	C14 Malignant neoplasm of other and ill-defined sites in the lip, oral cavity and pharynx (11.1%)		
Ch. II – Neoplasms in women (C00-D48)	*n* = 6	*n* = 313	*n* = 1194
	C67 Malignant neoplasm of bladder (16.7%)	C50 Malignant neoplasm of breast (13.4%)	C34 Malignant neoplasm of bronchus and lung (19.9%)
	C24 Malignant neoplasm of other and unspecified parts of biliary tract (16.7%)	C18 Malignant neoplasm of colon (9.3%)	C50 Malignant neoplasm of breast (13.0%)
	C26 Malignant neoplasm of other and ill-defined digestive organs (16.7%)	C34 Malignant neoplasm of bronchus and lung (8.6%)	C25 Malignant neoplasm of pancreas (8.0%)
	C22 Malignant neoplasm of liver and intrahepatic bile ducts (16.7%)		
	C57 Malignant neoplasm of other and unspecified female genital organs (16.7%)		
	C76 Malignant neoplasm of other and ill-defined sites (16.7%)		
Ch. VI – Diseases of the nervous system (G00-G99)	*n* = 35	*n* = 218	*n* = 325
	G30 Alzheimer disease (54.3%)	G40 Epilepsy (21.6%)	G30 Alzheimer disease (44.0%)
	G40 Epilepsy (37.1%)	G80 Cerebral palsy (19.3%)	G20 Parkinson disease (15.7%)
	G31 Other degenerative diseases of nervous system, not elsewhere classified (5.7%)	G30 Alzheimer disease (10.1%)	G12 Spinal muscular atrophy and related syndromes (12.0%)

ICD-10, International Classification of Diseases, Tenth Revision. The proportions presented in this table refer to the number of deaths related to a specific cause (3-digit ICD-10 code) divided by the total number of individuals who died within the disease category group (chapter code) as shown in each cell. In other words, the percentages refer to proportionate mortality within each chapter for each group. When ties of percentages were observed in the three leading causes of death, all causes with tie percentages were presented

### Mortality risk observed in groups

**Fig. 2:**
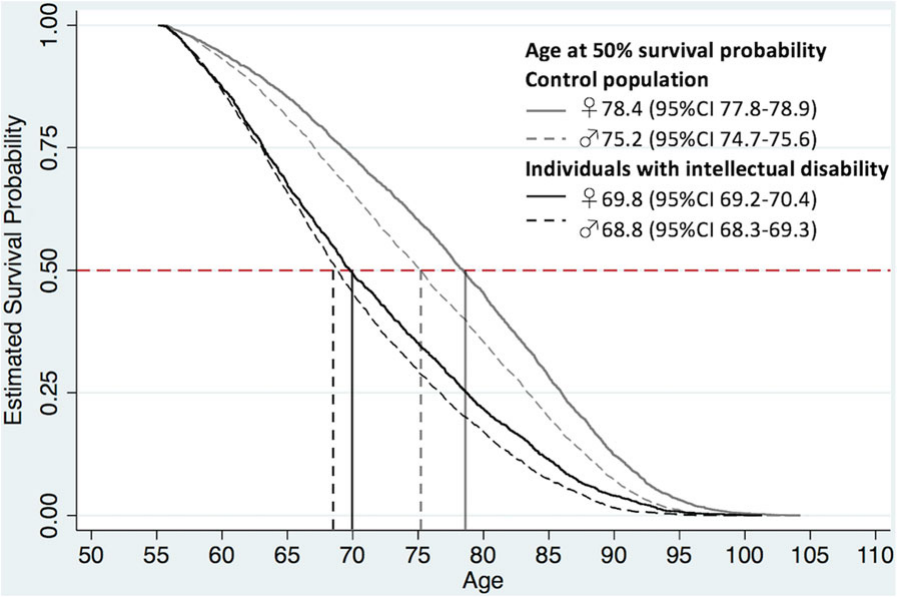
Survival curve for men and women with intellectual disability and the control population

Conditional Cox regression analyses showed that individuals with ID had a 4-fold higher mortality risk compared with the control population. When analysed for cause-specific mortality (Table 6), the mortality risk among individuals with ID ranged from 1.8 times higher for neoplasms to 12.5 times higher for diseases of the respiratory system compared with the control population. When the analyses were repeated to assess the hazard risk for men and women with DS, individuals with DS had much higher risk of death from respiratory- and nervous system-related diseases (note the large CIs for these two mortality groups). Figure 2 shows a lower survival rate among individuals with ID, with a median survival time of 68.8 years for men and 69.8 years for women with ID, compared with 75.2 years for men and 78.4 years for women in the control population.

**Table 6:** Hazard risk for mortality among individuals with intellectual disability; models run separately for all deaths and cause-specific deaths

	All individuals with intellectual disability vs. their control population (15,289 vs. 76,445 individuals)	Only individuals with Down’s syndrome vs. their control population (942 vs. 4710 individuals)
	Hazard Ratio (95% CI)	Hazard Ratio (95% CI)
All deaths	4.14 (3.98–4.31)	25.4 (21.3–30.4)
Circulatory diseases-related deaths	4.20 (3.92–4.50)	25.0 (17.7–35.4)
Neoplasm-related deaths	1.78 (1.63–1.94)	1.22 (0.70–2.16)
Respiratory diseases-related deaths	12.5 (10.9–14.2)	170 (84.6–345)
Nervous diseases-related deaths	6.68 (5.58–7.99)	41.2 (18.8–90.3)

## Discussion

To our knowledge, this is the first national longitudinal study mapping mortality patterns and risk of cause-specific death for first generation older individuals with ID who experienced the transition in living conditions following the closure of care institutions in Sweden. Our study reported survival and causes of death among individuals with ID aged 55 years and older in Sweden from 2002 to 2015.

### Survival among individuals with ID

In this study, we observed a 4-fold higher mortality rate and a shorter median survival time among older adults with ID compared with the general older adult population. Similar results have been reported in the US [29, 30], Canada [31], the UK [32], Finland [33] and Ireland [34]. Women in the control population and women with ID had higher life expectancies than men. The gender gap in median survival was relatively small among Swedish older adults with ID compared with the control population. Our finding of better survival among women with ID confirm those reported in the US [29] and Taiwan [35]. However, these findings differ from results reported in the UK [4, 36, 37], Finland [33] and Canada [31], which showed higher mortality rates among women with ID compared with their male counterparts. As our study focused on older adults (aged 55+ years), we might have missed individuals with more severe and profound ID, such as Rett syndrome, Williams syndrome and tuberous sclerosis, among whom life expectancies are reported with a maximum age of 50–59 years [1].

### Causes of death among individuals with ID

Our study confirmed observations from other countries that some causes of death were more common among individuals with ID [38]. Deaths from respiratory diseases (mainly pneumonia and pneumonitis due to solids/fluid) were more common among individuals with DS compared with the control population. A similar observation was reported by Englund et al., who identified pneumonia as the main cause of death among individuals with DS [3]. Among individuals with ID (DS excluded), cardiovascular deaths dominated, followed by neoplasm deaths. A national population-based study in the UK showed that adults with ID were more prone to die from congenital malformation and diseases in different body systems, including the nervous, respiratory, digestive, genitourinary and circulatory systems. However, their comparative risks of dying from respiratory and digestive organ neoplasms were lower compared with the general population [24]. Individuals with ID are more prone to cardiovascular disease risk factors, morbidity and mortality than the general population. A study in the Netherlands showed that hypertension, diabetes, hypercholesterolemia and metabolic syndrome were common health problems among adults with ID [39]. Cardiovascular disease health promotion and prevention programmes should be tailored for individuals with ID, to prevent or delay cardiovascular diseases and premature mortality in this vulnerable group.

Our study showed that deaths related to diseases of the nervous system (mainly epilepsy) were more prevalent among individuals with ID compared with the control population. Recent reviews showed a higher risk of sudden unexpected death in epilepsy among individuals with ID [40, 41]. A Swedish study using a national cohort of individuals with DS who died between 1969 and 2003 reported that death from dementia was common among individuals with DS [3]. A recent systematic review showed that pneumonia-related mortality increased more than 2-fold among individuals with dementia [42], which might explain the patterns of morbidity and mortality observed among individuals with DS in Sweden [3].

As the population ages, more people are living with dementia or Alzheimer’s disease. Our study verified existing evidence that dementia is a common cause of death among individuals with DS as well as in the general population. Dementia is an age-related degenerative disease and its prevalence increases sharply between ages 40–60 years among individuals with DS [43]. To date, no cure for dementia is available; however, treatments are available to slow the progression of dementia to more severe stages [44]. Early diagnosis of dementia will decrease the need for long-term care and improve the quality of life among people with dementia. Understanding how current health and social care systems provide dementia care for individuals with DS is necessary to develop best practice strategies and programmes [16].

Our study confirmed there were fewer deaths due to neoplasms among individuals with ID, especially among those with DS, compared with the control population. Breast cancer was the leading cause of death among women with ID (DS excluded) in our study. Neoplasms in women with ID were diagnosed in more advanced stages compared with women in the general population [45]. Barriers to accessing screening programmes have been reported at individual and health systems levels. A study in Canada highlighted the inequitable access to cancer screening among women with ID, with a lower rate of access to cervical neoplasms and mammography screening compared with the general population [46]. Attention should be directed to measures to reduce the barriers and increase access for individuals with ID, to ensure this population benefits from available screening programmes [46, 47]. Further studies should use longer-term longitudinal data to determine if the mortality risk related to neoplasms among individuals with ID has changed over time, and how access to cancer prevention and treatment programmes may influence mortality risk over time.

Individuals with ID, especially those with more severe forms of ID, are often dependent on others for care and assistance in activities of daily living, and have potential to be excluded from receiving high quality education and healthcare services [5, 48]. Barriers to care for individuals with ID include less interpretable symptoms of illness, more difficulty in performing examinations and communication problems [48]. The quality of care received by older individuals with ID is dependent on the skills of care staff in overcoming barriers in caring for individuals with ID, staff psychological factors that might influence interactions with individuals with ID and the quality of care delivered [49]. Future studies should investigate whether life-threatening diseases among individuals with ID with different living arrangements could have been averted. Early identification of symptoms and signs by trained and qualified carers in environments such as group homes means that seriously ill individuals with ID with life-threatening conditions may be referred to hospitals for adequate advanced care in a timely manner.

### Strengths

In this study, we identified a more representative population with ID by combining the LSS register and the NPR than what we would have achieved using each register independently. Even so, the true prevalence of individuals with ID in Sweden cannot be fully established. The LSS register contains an estimated 50–70% of adults with ID in Sweden, and includes all individuals with ID who live in group homes, work in day care centres or receive any other support from society [50]. To obtain a more reliable estimate of the prevalence of ID in Sweden, future studies should include other registers, such as the primary care register, and use a capture-recapture method [51]. No national-level register for primary health care in Sweden exists yet, as all registers are locally or regionally-based.

This study fills in the gap of knowledge on cause-of-death among older people with intellectual disabilities. Use of the Cause of Death Register allowed us to better identify the leading cause of death among individuals with ID. Disease diagnosis among individuals with ID may be challenging. Merrick et al. reported a lower prevalence of cardiovascular disease with increasing severity in cognitive dysfunction that could be explained by under-diagnosis [52]. The detailed information in the death register allowed us to understand the patterns of death among this population, which may contribute to better healthcare planning and management for individuals with ID. Considering the growing number of evidence on this field, a systematic review and/or meta-analysis on cause-of-death patterns among older people with intellectual disabilities is warranted.

### Limitations

As the Swedish population register does not contain any disease/diagnosis information, it is possible that the matched control population also included individuals with ID who did not access any LSS services or did not have an ID diagnosis recorded in the NPR. The LSS register might also include individuals without ID but with an autism diagnosis who received service and support under the LSS law. It is also possible that DS was not recorded as either the underlying or contributing cause of death. These potential misclassification biases might mean our results are under- or overestimated. However, we anticipated this by using clear inclusion criteria for ID and exclusion of atypical autism criteria.

We did not classify the causes of death by underlying and contributing causes of death. The focus was mainly on the underlying cause of death, which was imputed with the contributing cause of death only recorded for those who had been identified with DS as an underlying or contributing cause of death. There was also no detail regarding different ID diagnoses other than DS, which might have enriched our analyses and interpretation of cause-of-death data.

As the LSS data is only available from 2007 (the register started in 2004 with very limited data) and no earlier register of individuals with ID exists, it is not possible for us to control for prior institutionalisation among the individuals with ID before the closure of care institutions in the 70s. In Sweden, no register covering the adult age for the cohort of older people in our study exists. Such registration was forbidden considering what happened to people with intellectual disability before or during the World War II Period. The establishment of the LSS law and the LSS register in 2004 can therefore be seen as a breakthrough in understanding the health patterns and health care needs among people with intellectual disability in Sweden. Therefore, the results of this study should be interpreted in light of other potential confounding factors such as residential history, prior institutionalization, access to health care, exposure to different factors, as well as life-style behaviours which might influence the outcome of this study – the mortality. Similarly, as these data are not available in our study, we cannot explain whether the differential mortality level observed among individuals with ID and the general population were due to biological differences or the effects of the above-mentioned confounding factors.

## Conclusions

Our study shows an almost 4-fold higher mortality rate among older adults with ID in Sweden and a risk for respiratory disease deaths up to 13-fold higher. Older adults with ID also have a lower median survival time and shorter lifespan. The leading cause of death among individuals with DS is respiratory diseases (mainly pneumonia), whereas cardiovascular diseases are the leading cause of death among individuals with ID (DS excluded), as was also observed in the general population. Care for older adults with ID, particularly during the terminal stage of their illness, needs to be tailored based on the understanding of their main health problem.

## Additional file

Additional file 1: This file contains additional material which shows the mortality rate per 100,000 people for three leading causes of death within each selected major cause of death, based on ICD-10 chapters. (DOCX 15 kb)

## Abbreviations

CI: Confidence Interval
DS: Down syndrome
ICD-10: International Statistical Classification of Diseases and Related Health Problems, Tenth Revision
ID: Intellectual disability
LSS: The Act Concerning Support and Service for People with Certain Functional Impairments
NPR: National Patient Register
SOL: The Social Service Act
