# Supplementary material for: Mortality patterns and risk among older men and women with intellectual disability: a Swedish national retrospective cohort study
Source: BMC Geriatr. 2017 Nov 22;17:269. doi: 10.1186/s12877-017-0665-3 (PMC5700486; doi:10.1186/s12877-017-0665-3)
Supplement: Additional file 1: — This file contains additional material which shows the mortality rate per 100,000 people for three leading causes of death within each selected major cause of death, based on ICD-10 chapters. (DOCX 15 kb) [file 12877_2017_665_MOESM1_ESM.docx]

**Additional file 1: Mortality rate per 100,000 people for three leading causes of death within each selected major cause of death, based on ICD-10 chapters**

| **ICD categories** | **Underlying causes of death among individuals with DS**  **(n=761)** | **Underlying causes of death among individuals with ID (DS excluded)**  **(n=3,977)** | **Underlying causes of death among matched control population**  **(n=8,364)** |
| --- | --- | --- | --- |
| **Total person year** | 8,848 | 190,788 | 1,032,245 |
| **Ch. IX – Diseases of the circulatory system**  **(I00-I99)** | **n=122** | **n=1,368** | **n=2,959** |
|  | I50 Heart failure (350)  I21 Acute myocardial infarction (203)  I25 Chronic ischaemic heart disease (170) | I21 Acute myocardial infarction (167)  I25 Chronic ischaemic heart disease (119)  I50 Heart failure (85) | I21 Acute myocardial infarction (69)  I25 Chronic ischaemic heart disease (61)  I50 Heart failure (22) |
| **Ch. X – Diseases of the respiratory system**  **(J00-J99)** | **n=51** | **n=468** | **n=530** |
|  | J69 Pneumonitis due to solids and liquids (181)  J18 Pneumonia, organism unspecified (113)  J45 Asthma (45)  J20 Acute bronchitis (45)  J98 Other respiratory disorders (45) | J18 Pneumonia, organism unspecified (129)  J44 Other chronic obstructive pulmonary disease (49)  J69 Pneumonitis due to solids and liquids (25) | J44 Other chronic obstructive pulmonary disease (25)  J18 Pneumonia, organism unspecified (13)  J84 Other interstitial pulmonary diseases (4) |
| **Ch. II – Neoplasms in men**  **(C00-D48)** | **n=9** | **n=381** | **n=1,555** |
|  | D37 Neoplasm of uncertain or unknown behaviour of oral cavity and digestive organs (34)  C25 Malignant neoplasm of pancreas (11)  C67 Malignant neoplasm of bladder (11)  C16 Malignant neoplasm of stomach (11)  C90 Multiple myeloma and malignant plasma cell neoplasms (11)  C76 Malignant neoplasm of other and ill-defined sites (11)  C14 Malignant neoplasm of other and ill-defined sites in the lip, oral cavity and pharynx (11) | C18 Malignant neoplasm of colon (26)  C61 Malignant neoplasm of prostate (21)  C34 Malignant neoplasm of bronchus and lung (14) | C34 Malignant neoplasm of bronchus and lung (28)  C61 Malignant neoplasm of prostate (25)  C25 Malignant neoplasm of pancreas (10) |
| **Ch. II – Neoplasms in women**  **(C00-D48)** | **n=6** | **n=313** | **n=1,194** |
|  | C67 Malignant neoplasm of bladder (11)  C24 Malignant neoplasm of other and unspecified parts of biliary tract (11)  C26 Malignant neoplasm of other and ill-defined digestive organs (11)  C22 Malignant neoplasm of liver and intrahepatic bile ducts (11)  C57 Malignant neoplasm of other and unspecified female genital organs (11)  C76 Malignant neoplasm of other and ill-defined sites (11) | C50 Malignant neoplasm of breast (22)  C18 Malignant neoplasm of colon (15)  C34 Malignant neoplasm of bronchus and lung (14) | C34 Malignant neoplasm of bronchus and lung (23)  C50 Malignant neoplasm of breast (15)  C25 Malignant neoplasm of pancreas (9) |
| **Ch. VI – Diseases of the nervous system**  **(G00-G99)** | **n=35** | **n=218** | **n=325** |
|  | G30 Alzheimer disease (215)  G40 Epilepsy (147)  G31 Other degenerative diseases of nervous system, not elsewhere classified (23) | G40 Epilepsy (25)  G80 Cerebral palsy (22)  G30 Alzheimer disease (12) | G30 Alzheimer disease (14)  G20 Parkinson disease (5)  G12 Spinal muscular atrophy and related syndromes (4) |

**Note:** ICD-10, International Classification of Diseases, Tenth Revision. Numbers in brackets represent the cause-specific mortality rate (per 100,000 person years) for each cause, calculated by dividing the number of deaths related to each cause by the total person time for each group. When ties of number of deaths were observed in the leading causes of death, all causes with tie numbers were presented.
